# Assisted reproductive technology and interactions between serum basal FSH/LH and ovarian sensitivity index

**DOI:** 10.3389/fendo.2023.1086924

**Published:** 2023-05-03

**Authors:** Yumei He, Ling Liu, Fei Yao, Chenyu Sun, Muzi Meng, Yunzhu Lan, Chengliang Yin, Xingyu Sun

**Affiliations:** ^1^ Department of Gynecology, The Affiliated Traditional Chinese Medicine Hospital of Southwest Medical University, Luzhou, Sichuan, China; ^2^ Department of Reproductive Medicine Center, The Affiliated Hospital of Southwest Medical University, Luzhou, China; ^3^ Department of Thyroid and Breast Surgery, The Second Affiliated Hospital of Anhui Medical University, Hefei, China; ^4^ UK Program Site, American University of the Caribbean School of Medicine, Preston, United Kingdom; ^5^ Bronxcare Health System, New York City, NY, United States; ^6^ The Fourth Affiliated Hospital, Zhejiang University School of Medicine, Hangzhou, China; ^7^ Faculty of Medicine, Macau University of Science and Technology, Macau, Macao SAR, China

**Keywords:** FSH/LH, ovarian sensitivity index (OSI), anti-Mullerian hormone, pregnancy, assisted reproductive technology

## Abstract

**Objectives:**

This study aimed to investigate whether the FSH (follicle-stimulating hormone)/LH (Luteinizing hormone) ratio correlates with ovarian response in a cross-sectional retrospective study of a population with normal levels of anti-Müllerian hormone (AMH).

**Methods:**

This was a retrospective cross‐sectional study with data obtained from medical records from March 2019 to December 2019 at the reproductive center in the Affiliated Hospital of Southwest Medical University. The Spearmans correlation test evaluated correlations between Ovarian sensitivity index (OSI) and other parameters. The relationship between basal FSH/LH and ovarian response was analyzed using smoothed curve fitting to find the threshold or saturation point for the population with mean AMH level (1.1<AMH<6μg/L). The enrolled cases were divided into two groups according to AMH threshold. Cycle characteristics, cycle information and cycle outcomes were compared. The Mann-Whitney U test was used to compare different parameters between two groups separated by basal FSH/LH in the AMH normal group. Univariate logistic regression analysis and multivariate logistic regression analysis were performed to find the risk factor for OSI.

**Results:**

A total of 428 patients were included in the study. A significant negative correlation was observed between OSI and age, FSH, basal FSH/LH, Gn total dose, and Gn total days, while a positive correlation was found with AMH, AFC, retrieved oocytes, and MII egg. In patients with AMH <1.1 ug/L, OSI values decreased as basal FSH/LH levels increased, while in patients with 1.1<AMH<6 ug/L, OSI values remained stable with increasing basal FSH/LH levels. Logistic regression analysis identified age, AMH, AFC, and basal FSH/LH as significant independent risk factors for OSI.

**Conclusions:**

We conclude that increased basal FSH/LH in the AMH normal group reduces the ovarian response to exogenous Gn. Meanwhile, basal FSH/LH of 3.5 was found to be a useful diagnostic threshold for assessing ovarian response in people with normal AMH levels. OSI can be used as an indicator of ovarian response in ART treatment.

## Introduction

In recent years, infertility, which affects human development and health, has become a global medical and sociological problem (1). Assisted reproductive technologies (ART) have been developed for more than 40 years. In recent decades, assisted reproduction techniques have evolved. However, even when good quality embryos are selected for transfer to the uterus, the implantation rate remains low. Sunderam et al. showed that despite a gradual increase in clinical pregnancy rates among infertile women treated with ART over the past decades, the live birth rate per *in vitro* fertilization-embryo transfer (IVF-ET) was only 38.1% (2). Fertility practitioners should be fully aware of the failure of IVF cycles to improve the success rate of ART. Controlled ovarian hyperstimulation (COH) is critical to the success of IVF-ET (3). However, COH can lead to two adverse outcomes (high ovarian response or low ovarian response) due to the different ovarian responses to COH (4). Accurate prediction of ovarian response is critical to improve *in vitro* fertilization (IVF) or intracytoplasmic sperm injection (ICSI) (5). Currently, there are no relevant informative markers that directly predict ovarian response. The ovarian response is predicted based on the assessment of ovarian reserve indicators (6).

Anti-Müllerian hormone (AMH) levels are positively correlated with follicle number and decrease with increasing age and decreasing follicle number. AMH levels are constant throughout the menstrual cycle and its serum levels are not affected by FSH, LH, and E2 levels. These unique characteristics make AMH a good predictor of ovarian reserve (7). In addition, many studies have shown that age, AMH levels and antral follicle count (AFC) may be predictors of ovarian response (8). However, in clinical practice, the above parameters may not always be evaluated satisfactorily and accurately, and there is a need for more reliable factors to evaluate ovarian reserve. Several potential indicators of ovarian function are influenced by both cyclic variability and aging, and both factors must be taken into account in assessing ovarian function, which makes interpretation a challenge. Du et al. have demonstrated no factors can unconditionally assess ovarian reserve (9). Although AMH and AFC are widely considered as ovarian markers, they do not correctly detect hyporesponsive patients with normal ovarian reserve markers (10–12). A study related to the basal FSH/LH ratio predicting *in vitro* fertilization outcome showed that the basal FSH/LH was associated with poor outcome of *in vitro* fertilization treatment and may be a predictor of decreased ovarian reserve (13).

It has been observed that both the absolute number of oocytes retrieved and total gonadotrophin dose are essential measures of ovarian responsiveness, and the ratio of the two is a better representation of ovarian responsiveness than either parameter alone.

Ovarian sensitivity index (OSI), was first proposed by Biasoni et al. (14). OSI has been found correlated to AMH and AFC, which have been suggested as predictors of ovarian responsiveness (15, 16). Using OSI as a measure of ovarian responsiveness would be better than the number of retrieved oocytes for different gonadotrophin dosages applied to different subjects daily. Pan et al. showed that when OSI values were low, ovarian sensitivity was lower and pregnancy rates were lower; when OSI values were high, the incidence of OHSS was higher and pregnancy rates were lower (17). Huber et al. showed that an OSI below 1.7 was considered a low ovarian response (18). We defined OSI as the number of retrieved oocytes/the total dose of administered gonadotrophins. The use of gonadotropins for ovulation induction is related to a variety of factors, including the patient’s age, body mass index (BMI), ovarian function, hormone levels, personal and family history, and the patient’s personal preferences and treatment goals. The use of gonadotropins may also be influenced by the patient’s lifestyle and environmental factors. Therefore, when using gonadotropins for ovulation induction, these factors should be considered to ensure the treatment’s effectiveness and safety. Therefore, searching for new accurate, safe and effective markers is very important.

In the present study, we focused our research mainly on normal AMH population. The study aims were: (1) to detect the association between ovarian sensitivity index (OSI) and varieties of ovarian reserve, (2) to examine whether serum basal FSH/LH is corelated to OSI, (3) to assess whether OSI affects ovarian response, and (4) to find the threshold/saturation point in the study population.

## Methods

### Patients enrollment

In the cross-sectional retrospective study, infertile women underwent IVF/ICSI treatment at the reproductive center in the Affiliated Hospital of Southwest Medical University between March 2019 and December 2019.

#### Inclusion criteria

(1) Aged<40 years;
(2) FSH < 25 U/L;
(3) Patients received IVF/ICSI treatment;
(4) Complete case information.

#### Exclusion criteria

(1) Patients with polycystic ovarian syndrome (PCOS), endometriosis, premature ovarian insufficiency (POI);
(2) Patients with a high incidence of ovarian stimulation hyperresponsiveness;
(3) Patients with a history of ovarian tumors and other malignancies;
(4) Patients with a history of endocrine abnormalities such as diabetes, hyperthyroidism, and hypothyroidism.

### Data collection

Collected data included age, duration of infertility, BMI, AFC, AMH, basal FSH, basal LH, basal estradiol (E2) and basal P (progesterone), total Gn dose, total Gn days, oocytes recovered, number of embryos transferred, number of MII eggs, HCG day E2 level, HCG day LH level and HCG day P level.

### Ovarian sensitivity index calculation

Ovarian sensitivity index (OSI) was calculated by the following formula: OSI= Retrieved oocytes×1000/total Gn doses

### Hormone detection and analyses

Venous blood was collected into plain serum tubes and all samples were centrifuged (2–8°C, 2,000 g, 10 min) within 1 h of blood collection to separate the serum. In order to separate serum from venous blood, all samples were centrifuged (2–8°C, 2,000 g, 10 minutes) within 1 h of blood collection. Each aliquot from each patient was evaluated in random order in the same run, and all hormones were analyzed simultaneously. Each hormone was measured with an Elecsys^®^ assay in conjunction with a cobas e 601 module of a cobas^®^ 6000 analyzer (Roche Diagnostics, Mannheim, Germany) according to the producer’s instructions.

### Ovulation induction

All patients received the same ovulation promotion protocols, using the same hormones and the same dose adjustment criteria. Ovulation was induced using antagonists or long-term protocols. Recombinant follicle-stimulating hormone (rFSH, Gonal-F, Merck-Serono, Brazil) was given daily on day 2 of the menstrual cycle as the start of the antagonist protocol. The dose of rFSH was adjusted according to the ovarian response measured by estradiol serum concentrations, and follicular growth was monitored by vaginal ultrasound. When follicles reached 14 mm, patients started receiving gonadotropin-releasing hormone (GnRH) antagonists (Cetrotide, MerckSerono, Brazil) associated with rFSH. For the long-term regimen, treatment began with subcutaneous administration of 3.75 mg of GnRH agonist (Gonapeptyl, Ferring, Brazil) on day 21 of their menstrual cycle to suppress pituitary function. To confirm the downregulation of estradiol, serum estradiol concentrations and vaginal ultrasonography were performed approximately 10 days later. If the estradiol concentration was <30 pg/ml and ultrasound showed an endometrial thickness of <3 mm, patients were considered ready to start ovulation induction. After confirmation of suppression, patients received daily doses of rFSH for ovulation induction. In both regimens, oocyte maturation was induced with recombinant human chorionic gonadotrophin (hCG, Ovidrel, Merck-Serono, Brazil) when at least two follicles reached a mean size of 17 mm with concordant estradiol levels (approximately 200 pg/ml).

### Embryo transfer technique

All embryo transfers were performed under ultrasound control. Therefore, patients were asked to fill their bladders to provide an acoustic window for uterine visualization. The catheter tip (Wallace, Smits-Medical, Dublin, Ireland) was placed 1.0–2.0 cm below the apex of the uterine cavity. Avoiding uterine contractions, a pipette was inserted slowly from the cervical os into the uterine cavity until it reached the fundus uteri.

### Outcome measure

The pregnancy diagnosis was made by a positive hCG test on Day 14 after embryo transfer. The patient underwent transrectal ultrasonography to monitor the gestational sac and the clinical pregnancy diagnosis was confirmed on day 28 post-transfer. Luteal phase support was continued until 12 weeks of gestation. The ratio of basal FSH/LH was computed to detect the turning point of OSI.

### Statistical analysis

SPSS-22.0 software (SPSS Inc. Chicago, IL, USA) was used for statistical analysis. Continuous variables were expressed as median scores and compared using the Mann-Whitney U test. Categorical variables were applied as percentages and compared using Fisher’s exact test. Median [P25%, P75%] and Mann-Whitney U tests were used to represent and compare continuous variables. The t-test (2-tailed) was used for comparison between groups of measures, and the Kruskal-Wallis test was used when normality was not satisfied for comparison between groups. Enumeration data were expressed as percentages using the χ^2^ test. The Spearman correlation coefficient was applied to explore the correlation between variables. Differences were considered statistically significant at a P-value < 0.05. An additional logistic regression analysis was performed, and the outcome was a binary OSI variable obtained using the detected turning point as cutoff, which differs from the continuous OSI mentioned elsewhere.

## Results

### General characteristics of this study

**Figure 1:**
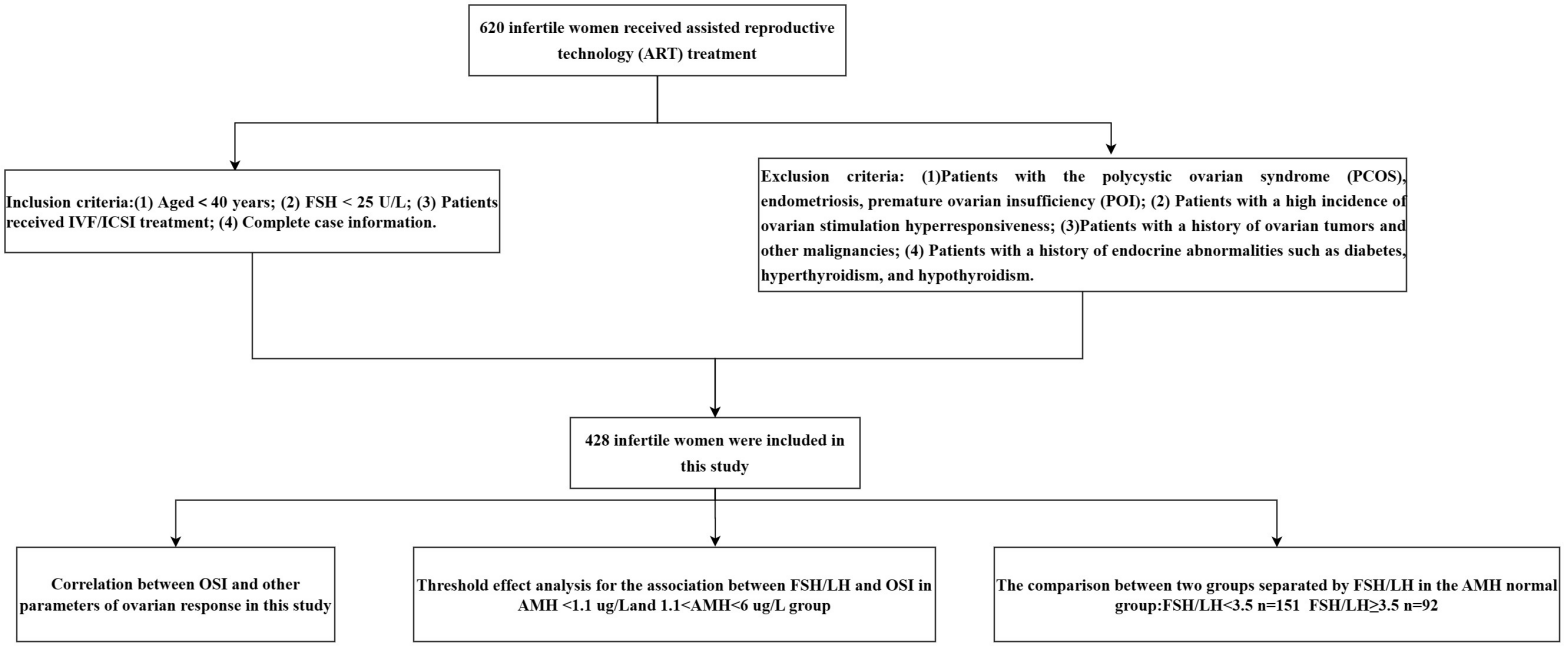
The flow chart of this research.

Four hundred twenty-eight patients who met the selection criteria were included in this study. **Figure 1** shows the study procedure flowchart. Patient information included in this study is shown in **Table 1**. The median age of the patients was 31 years and the median duration of infertility was 3 years. The median AMH and AFC are 4.06 ug/L and 8, respectively. The clinical pregnancy rate in this study was 28%. Additional patient information is shown in **Table 1**.

**Table 1:** Baseline characteristics of the patients enrolled.

Parameters		Value
Age(years)		31(28-34)
Infertility duration (years)		3(2-5)
Body mass index (BMI, kg/m^2^)		22.03(19.81-24.65)
AFC(pieces)		8(6-9)
Anti-Müllerian hormone (AMH, ug/L)		4.06(2.25-7.55)
Basal Follicle-stimulating hormone(FSH, U/L)		8.57(7.26-10.39)
Basal Luteinizing hormone(LH, mU/L)		3.15(2.21-4.65)
basal E2(ng/L)		41.88(32.50-59.50)
basal P(ug/L)		0.61(0.39-0.97)
Gn total dose(U)		2400(1800-3000)
Gn total days (days)		11(9-13)
Retrieved oocytes(pieces)		9(6-13)
No. of transferred embryos(pieces)		2(1-2)
No. of MII eggs (pieces)		8(5-11)
E_2_ level on HCG day(ng/L)		2706.46(1671.46-3370.23)
LH level on HCG day(mU/L)		0.81(0.46-1.36)
P level on HCG day(ug/L)		0.79(0.55-1.08)
Outcomes	No pregnancies	308(71.9%)
	Pregnancies	120(28%)

BMI, body mass index; AFC, antral follicle count; AMH, anti-Müllerian hormone; FSH, Follicle-stimulating hormone; LH, Luteinizing hormone; E_2_, estrogen; Gn, gonadotropin; Values are expressed as Medians [P25%, P75%].

### The correlation between OSI and other parameters

**Figure 2:**
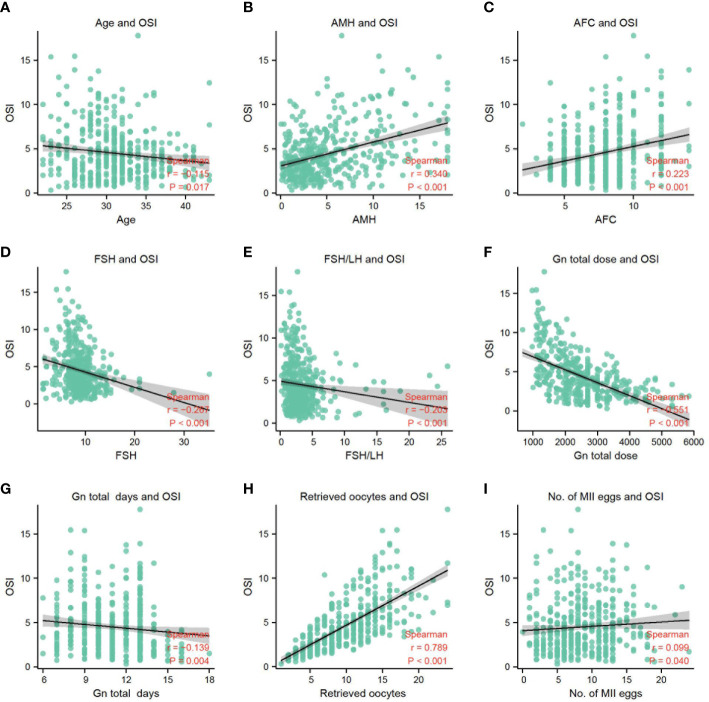
**(A)** The correlation between OSI and age. **(B)** The correlation between OSI and AMH. **(C)** The correlation between OSI and AFC. **(D)** The correlation between OSI and FSH. **(E)** The correlation between OSI and FSH/LH. **(F)** The correlation between OSI and Gn total dose. **(G)** The correlation between OSI and Gn total days. **(H)** The correlation between OSI and retrieved oocytes. **(I)** The correlation between OSI and No. of MII eggs.

The results of the correlation analysis between OSI and other parameters in this study are shown in **Table 2**. There was a significant negative correlation as follows, for OSI with Age (*r_s_* =-0.115, *p*=0.017) (**Figure 2A**), FSH (*r_s_* =-0.267, *P*<0.001) (**Figure 2D**), basal FSH/LH (*r_s_* =-0.203, *P*<0.001) (**Figure 2E**), Gn total dose (*r_s_* =-0.551, *P*<0.001) (**Figure 2F**), and Gn total days (*r_s_* =-0.319, *P*=0.004) (**Figure 2G**). There is also a significant positive correlation as follows, for OSI with AMH (*r_s_* = 0.340, *P*<0.001) (**Figure 2B**), AFC(*r_s_* =0.223, *P*<0.001) (**Figure 2C**), Retrieved oocytes (*r_s_* =0.789, *P*<0.001) (**Figure 2H**) and MII egg (*r_s_* =0.099, *P*=0.040) (**Figure 2I**). More detailed results were shown in **Table 2**.

**Table 2:** Correlation between OSI and other parameters of ovarian response in this study.

Parameter	Ovarian sensitivity index	
	Correlation coefficient	*p*-value
Ages(years)	-0.115	0.017
Infertility duration(years)	-0.044	0.362
Body mass index (BMI, kg/m^2^)	-0,043	0.374
basal E2(ng/L)	0.078	0.107
basal P(ug/L)	0.004	0.927
Anti-Müllerian hormone (AMH, ug/L)	0.340	P<0.001
Antral follicle count (AFC)	0.223	P<0.001
Basal Follicle-stimulating hormone(FSH, U/L)	-0.267	P<0.001
Basal Luteinizing hormone(LH, mU/L)	0.057	0.238
FSH/LH	-0.203	P<0.001
Gn total dose(IU)	-0.551	P<0.001
Gn total days (d)	-0.139	0.004
Retrieved oocytes(pieces)	0.789	P<0.001
No. of transferred embryos(pieces)	-0.037	0.448
No. of MII eggs (pieces)	0.099	0.040
E_2_ level on HCG day(ng/L)	0.032	0.512
LH level on HCG day(mU/L)	-0.041	0.397
P level on HCG day(ug/L)	-0.134	0.006

BMI, body mass index; AFC, antral follicle count; AMH, anti-Müllerian hormone; FSH, Follicle-stimulating hormone; LH, Luteinizing hormone; E_2_, estrogen, E_2_;Gn, gonadotropin; Statistically significant(P<0.05,P<0.001).

Models I, linear analysis; Models II, non-linear analysis. LRT test, Logarithmic likelihood ratio test (p value<0.05 means Models II is significantly different from Models I, which indicates a non-linear relationship). Adjusted: adjusted for age, BMI, AMH, E2, and AFC; BMI, body mass index: AMH, anti-Müllerian hormone; E_2_, estrogen, E_2;_ AFC, antral follicle count; OSI, Ovarian sensitivity index.

### The relationship between basal FSH/LH and ovarian response in AMH <1.1 ug/L and 1.1<AMH<6 ug/L groups

**Figure 3:**
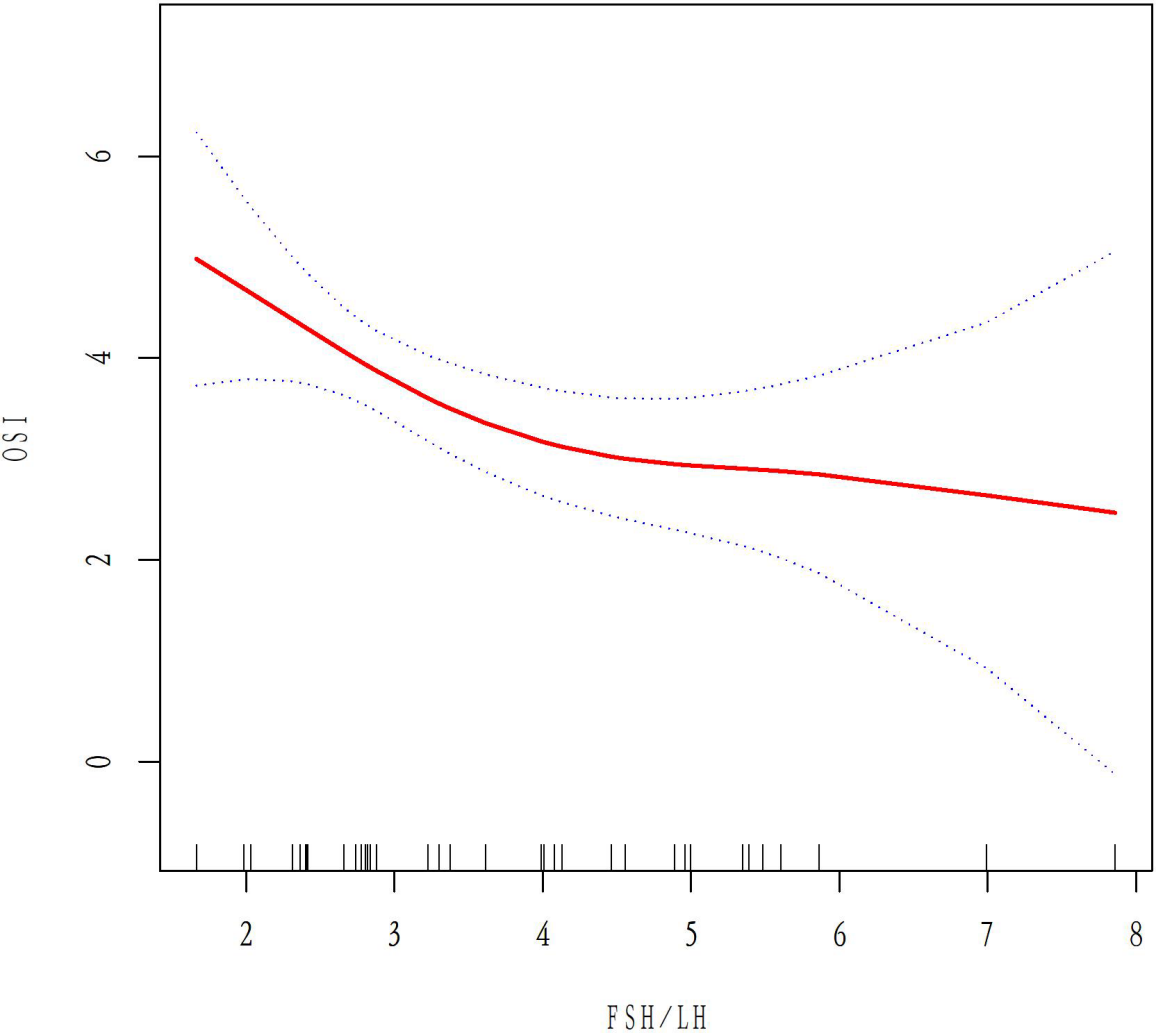
Relationship between basal FSH/LH levels and OSI values in the AMH<1.1ug/L group.

**Figure 4:**
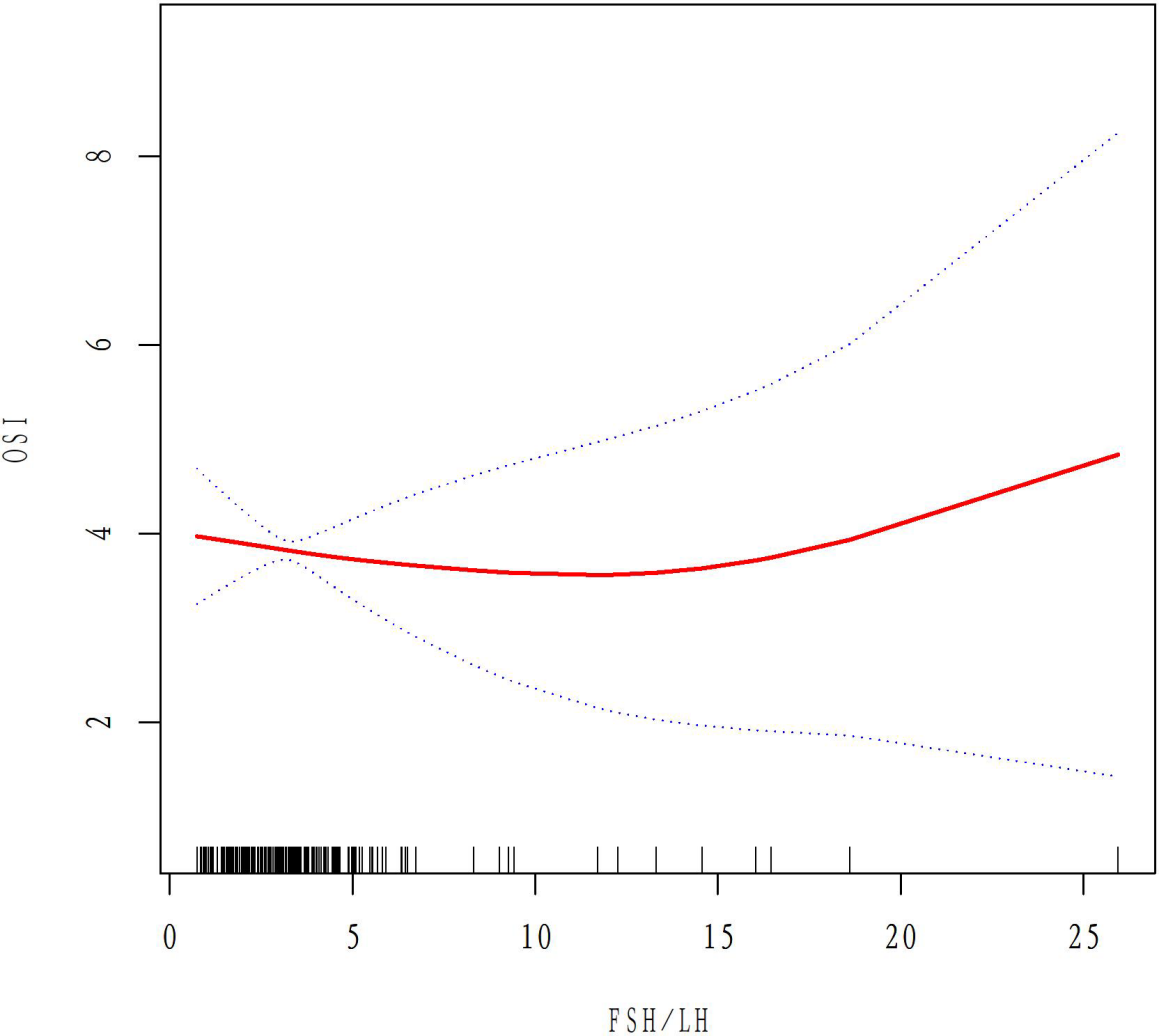
Relationship between basal FSH/LH levels and OSI values in the1.1<AMH<6ug/L group.

A total of 50 patients with AMH < 1.1ug/L and 243 patients with 1.1<AMH<6ug/L were analyzed to examine the relationship between basal FSH/LH and OSI while excluding ovarian response-related factors such as age, BMI, AMH, E2, and AFC. In the AMH<1.1ug/L group, OSI values decreased as basal FSH/LH levels increased (**Figure 3**). In contrast, for the 1.1<AMH<6ug/L group, OSI values remained stable and the curve was smooth with increasing basal FSH/LH levels (**Figure 4**). **Table 3** (revised) presents the threshold effect analysis for the association between FSH/LH and OSI in two groups with different AMH levels: AMH <1.1 ug/L and 1.1<AMH<6 ug/L. The table is divided into two sections, with one section for each group. Each section contains two models (Models I and II) and their respective adjusted beta coefficients (95% CI) and P-values. In Model I (linear analysis) for the group with AMH <1.1 ug/L, the one-line slope has an adjusted beta coefficient of -0.3 with a 95% CI of (-0.9, 0.3) and a P-value of 0.413. For the group with 1.1<AMH<6 ug/L, the one-line slope has an adjusted beta coefficient of -0.1 with a 95% CI of (-0.2, 0.1) and a P-value of 0.358. In Model II (non-linear analysis), a turning point is identified for each group. For the group with AMH <1.1 ug/L, the turning point is 2.3, with a slope1 of 2.1 (95% CI: -4.4, 8.7) and P-value of 0.513 for values below 2.3, and a slope2 of -0.1 (95% CI: -0.3, 0.3) and P-value of 0.316 for values above 2.3. For the group with 1.1<AMH<6 ug/L, the turning point is 3.5, with a slope1 of -0.2 (95% CI: -0.6, -0.1) and P-value of 0.049 for values below 3.5, and a slope2 of 0.1 (95% CI: -0.1, 0.3) and P-value of 0.052 for values above 3.5. The LRT test results indicate that there is a significant difference between Models I and II for both groups, with P-values of 0.03 for the AMH <1.1 ug/L group and 0.042 for the 1.1<AMH<6 ug/L group, suggesting a non-linear relationship between FSH/LH and OSI in both groups.

**Table 3:** Threshold effect analysis for the association between FSH/LH and OSI in AMH <1.1 ug/L and 1.1<AMH<6 ug/L group.

Models	OSI (group: AMH<1.1 ug/L)		Models	OSI (group:1.1<AMH<6ug/L)	
	Adjustedβ (95%CI)	*P*-valve		Adjustedβ(95%CI)	*P*-valve
**Models I**			**Models I**		
One line slope	-0.3(-0.9,0.3)	0.413	One line slope	-0.1(-0.2,0.1)	0.358
**Models II**			**Models II**		
Turning point	2.3		Turning point	3.5	
< 2.3 slope1	2.1(-4.4,8.7)	0.513	<3.5 slope1	-0.2(-0.6,-0.1)	0.049
>2.3 slope2	-0.1(-0.3,0.3)	0.316	>3.5 slope2	0.1(-0.1,0.3)	0.052
**LRT test**		0.03	**LRT test**		0.042

Models I, linear analysis; Models II, non-linear analysis. LRT test, Logarithmic likelihood ratio test (p value<0.05 means Models II is significantly different from Models I, which indicates a non-linear relationship). Adjusted: adjusted for age, BMI, AMH, E2, and AFC; BMI: body mass index: AMH, anti-Müllerian hormone; E2, estrogen, E2; AFC, antral follicle count; OSI, Ovarian sensitivity index.

### The comparison between two groups separated by basal FSH/LH in the AMH normal group

The results comparing age, infertility duration, BMI, AFC, AMH, and FSH (basal FSH/LH<3.5 and basal FSH/LH≥3.5) in the two groups are shown in **Table 4**. The following variables were statistically significant: Age, BMI, AFC, AMH, FSH, LH, E2, total Gn dose, and retrieved oocytes. Age, BMI, FSH, and total Gn dose were significantly higher in the basal FSH/LH≥3.5 group than in the basal FSH/LH<3.5 group (*P*<0.05). AFC, AMH, LH, and E2 were significantly lower in the basal FSH/LH≥3.5 group than in the basal FSH/LH<3.5 group (*P*<0.05). Although the pregnancy rate was also significantly higher than in the basal FSH/LH<3.5 group, the difference in pregnancy rate was not statistically significant(*P*=0.66). The retrieved oocytes, MII eggs, E_2_, and P on HCG day were lower than those in the basal FSH/LH<3.5 group (P<0.05).

**Table 4:** The comparison between two groups separated by FSH/LH in the AMH normal group.

Parameter	FSH/LH<3.5 n=151	FSH/LH≥3.5 n=92	*P-value*
Age(years)	31(28-34)	32(29-35)	0.042
Infertility duration (years)	3(2-5)	3(2-5)	0.265
Body mass index (BMI, kg/m^2^)	21.33(19.53-24.22)	21.77(19.82-24.65)	0.024
Antral follicle count (AFC)	8(6-9)	7(6-8)	0.049
Anti-Müllerian hormone (AMH, ug/L)	3.36(2.26-4.80)	3.05(2.39-4.02)	0.039
Follicle-stimulating hormone(FSH, U/L)	8.28(7.37-10.06)	9.78(7.88-11.24)	0.001
Luteinizing hormone(LH, mU/L)	3.68(2.67-4.97)	2.13(1.28-2.56)	P<0.001
basal E2(ng/L)	47.03(34.40-64.94)	41.24(31.13-62.37)	0.031
basal P(ng/L)	0.67(0.38-1.08)	0.61(0.43-1.01)	0.417
Gn total dose	2475 (2025.00-2475)	2700 (2050-3375)	0.024
Gn total days	11(9-13)	11(9-13)	0.877
Retrieved oocytes	9(5-13)	8(6-12)	0.038
mature eggs	1(0.83-1.00)	1(0.88-1.00)	0.900
No. of transferred embryos(pieces)	2(1-2)	2(1-2)	0.805
No. of MII eggs (pieces)	8(6-11)	7(5-10)	0.043
E_2_ level on HCG day(ng/L)	2758.42(1863.22-3326.22)	2386.82(1473.28-3370.23)	0.045
LH level on HCG day(mU/L)	0.80(0.46-1.34)	0.94(0.56-1.18)	0.054
P level on HCG day(ug/L)	0.83(0.55-1.19)	0.81(0.58-1.23)	0.047
outcomes (Pregnancies %)	41(27.15%)	28(30.43%)	0.66

BMI, body mass index; AFC, antral follicle count; AMH, anti-Müllerian hormone; FSH, Follicle-stimulating hormone; LH, Luteinizing hormone; E_2_, estrogen, E_2_;Gn, gonadotropin; Statistically significant(P<0.05,P<0.001).

### Logistics regression analysis of OSI risk factors

**Figure 5:**
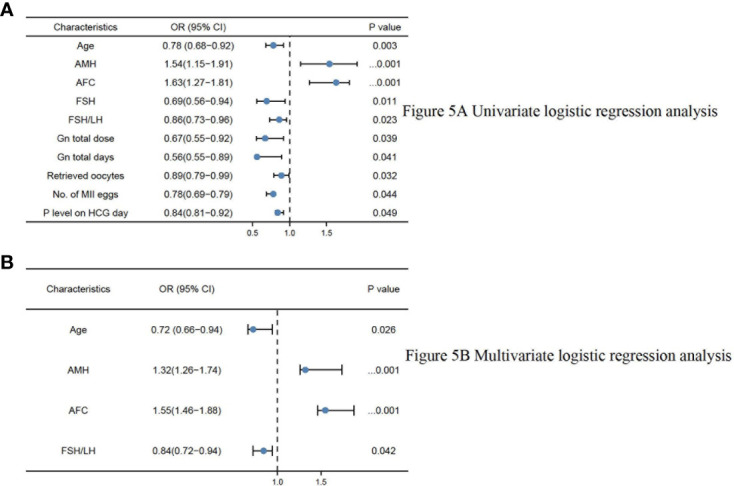
**(A)** Univariate logistic regression analysis. **(B)** Multivariate logistic regression analysis.

In the univariate and multivariate logistic regression analyses, there were finally four parameters significantly correlated with OSI (**Table 5**), namely age (odds ratio (OR) 0.72, 95% CI 0.66–0.94, P = 0.026), AMH (odds ratio (OR): 1.32, 95% CI 1.26–1.74, P<0.001), AFC (odds ratio (OR): 1.55, 95% CI 1.46–1.88, P<0.001), and basal FSH/LH (odds ratio (OR): 0.84, 95% CI 0.72–0.94, P=0.042). The logistics regression model showed Age, AMH, AFC, FSH, and basal FSH/LH were independent risk factors of OSI (P<0.05 for all, shown in **Table 5**; **Figures 5A, B**).

**Table 5:** Risk factors for OSI* identified by univariate logistic regression analysis and multivariate logistic regression analysis.

Characteristics	Univariate logistic regression analysis			Multivariate logistic regression analysis		
	OR	95%CI	P	OR	95%CI	P
Age	0.78	0.68-0.92	0.003	0.72	0.66-0.94	0.026
AMH	1.54	1.15-1.91	<0.001	1.32	1.26-1.74	<0.001
AFC	1.63	1.27-1.81	<0.001	1.55	1.46-1.88	<0.001
FSH	0.69	0.56-0.94	0.011	–	–	–
FSH/LH	0.86	0.73-0.96	0.023	0.84	0.72-0.94	0.042
Gn total dose	0.67	0.55-0.92	0.039	–	–	–
Gn total days	0.56	0.55-0.89	0.041	–	–	–
Retrieved oocytes	0.89	0.79-0.99	0.032	–	–	–
No. of MII eggs	0.78	0.69-0.79	0.044	–	–	–
P level on HCG day	0.84	0.81-0.92	0.049	–	–	–

AFC, antral follicle count; AMH, anti-Müllerian hormone; FSH, Follicle-stimulating hormone; LH, Luteinizing hormone. OSI*, a binary variable obtained using the detected turning point as cutoff.

## Discussion

*In vitro* fertilization (IVF) and intracytoplasmic sperm injection (ICSI) in Assisted reproductive technology (ART) are effective methods for the treatment of infertile women (19). However, the response to exogenous gonadotropins (Gn) may vary between women undergoing controlled ovarian hyperstimulation (COH), which is associated with patient prognosis, including cycle cancellation rate, exogenous gonadotropin dose, and pregnancy outcome (20). Poor ovarian response (POR) is an important issue in clinic infertility treatment. Even when appropriate ovarian stimulation is given, the prognosis for poor ovarian response (POR) remains unfavorable pregnancy outcome (21). Fertility declines gradually with age in women, starting to decline significantly around the age of 32 years and accelerating significantly after the age of 37 years (22). Screening for individuals and groups at risk of declining fertility is critical. In clinical practice, it is crucial to identify patients at risk of low ovarian response, and individualized ovulatory treatment for different ovarian responses may improve clinical pregnancy rates in infertile patients.

Reduced ovarian reserve is the dominant factor for poor ovarian response, and clinical indicators reflecting ovarian reserve include age, AMH AFC basic FSH level, and other relevant indicators (23). However, the evaluation of ovarian response is unsatisfactory and even the results can be inaccurate (24). Some new indicators such as AFC/TOC and FSH/LH may be a recent approaches in treating ovarian stimulation on COH therapy (10, 25). In early clinical practice, basic FSH was often used as an index to assess ovarian reserve, but ovarian response has been found to be lower in patients with normal FSH (26). The AFC is susceptible to human factors, resulting in a lack of accuracy and objectivity. In recent years, the combined use of AMH and AFC has allowed the assessment ovarian reserve. Patients with the low response, normal or high to exogenous Gn, can be identified by AFC and AMH (27). Mutlu et al. has shown that AMH is less sensitive in predicting low ovarian response (28). Overall, there are no specific markers to evaluate ovarian reserve and response independently, and a combined application for evaluation is still needed. Basal FSH/LH reflects ovarian response to exogenous Gn and is also associated with the length of the menstrual cycle prior to IVF/ICSI-ET (29). Kofinas et al. showed that elevated basal FSH/LH ratio >3 was more likely to result in individual menstrual cycle cancellation (15 vs 5.24%; p = 0.0001) in a total of 676 patients in the USA involved (30). Seckin et al. demonstrated that older women with a high basal FSH/LH (n = 23) had a significantly lower number of good grade embryos transferred (p = 0.04) and a significantly lower pregnancy rate (p = 0.03) compared to older women with a low basal FSH/LH ratio. However, in younger women, treatment outcomes were similar in both subgroups (31). Thus, they concluded that basal FSH/LH ratio is useful in predicting IVF outcomes in older women but does not appear to be an accurate predictor in younger women.

Patients with normal serum AMH levels but low ovarian response still exist and are easily overlooked by clinicians in clinical practice. In this study, we found that AMH and AFC decreased with increasing basal FSH/LH with increasing age by analyzing the normal AMH group. Therefore, we believe that the basal FSH/LH levels can reflect the reserve function of ovaries to some extent. Also, patients with elevated basal FSH/LH levels had higher total Gn doses but significantly fewer MII eggs than those with low basal FSH/LH levels in this study. Thus, patients with elevated basal FSH/LH levels had reduced sensitivity to exogenous Gn and reduced ovarian response. Previous studies found that the number of mature oocytes was reduced in those with elevated basal FSH/LH (23) levels and suggested that elevated basal FSH/LH levels were associated with a decreased final pregnancy rate (13). However, Arat et al. confirmed basal FSH/LH levels were not associated with the final cycle outcome (23) and that age and number of embryos transferred were independent factors affecting the final live birth rate (30). In the present study, we found no significant reduction in the number of mature eggs, number of embryos transferred, and final pregnancy rate in the population with basal FSH/LH ≥3.5. Therefore, we concluded that the number of mature eggs and the number of embryos transferred were not related to the level of basal FSH/LH. There was no significant difference in the number of mature eggs and final cycle outcomes. However, due to the small sample size, further follow-up is needed to calculate the cumulative pregnancy rate to determine whether the pregnancy outcome is affected by basal FSH/LH. In the present study, we found a decreasing trend in LH levels from the basal FSH/LH<3.5 group to the basal FSH/LH>3.5 group. We can further speculate that the decrease in ovarian response to exogenous Gn may be related to the increase in FSH and the decrease in LH level. A study found that a decrease in the basal LH level on the third day of the menstrual cycle reduced the number of retrieved oocytes and decreased the risk of hyperstimulation syndrome (OHSS) (32). Also, a decrease in LH may lead to a decrease in the number of antral follicles (33), as studied at the genetic level in rats. Noel et al. also demonstrated a reduced requirement for exogenous Gn during COH (34) in individuals with elevated endogenous LH levels to a certain extent. A complex interaction of molecular pathways occurs between female and male gametes during clinical pregnancies and live births. Olszewska et al. have demonstrated the relationship between methylation (5mC) and hydroxymethylation (5hmC) in sperm DNA concerning sperm chromatin protamination in three subpopulations of fertile normozoospermic controls and infertile patients with oligo-/oligoasthenozoospermia (35). Furthermore, Giebler et al. showed that PIWI-LIKE 1 and 2 transcript levels in the spermatozoa of the swim-up fraction were positively correlated with each other by analyzing how PIWI-LIKE 1-4 mRNA expression in ejaculated spermatozoa predicts outcomes of assisted reproductive techniques (ART), evaluating swim-up spermatozoa used for fertilization from 160 *in vitro* fertilization (IVF) or intracytoplasmic sperm injection (ICSI) cycles (36). In conclusion, our study sheds light on the potential impact of basal FSH/LH levels on ovarian response and ART outcomes, but it is essential to recognize the multifactorial nature of infertility and the diverse molecular pathways that come into play during the process of fertilization and embryo development. By expanding our knowledge in this area and exploring additional factors such as sperm DNA methylation and PIWI-LIKE transcript levels, we may be able to develop a more comprehensive understanding of infertility and improve the prognosis and treatment options for infertile couples seeking assistance through ART.

This study has the advantage of focusing on a specific population in Southwest China, an area that may not be economically developed but has an increasing trend of infertility patients. Additionally, these measurements were made in the same laboratory using the same equipment. As a result, laboratory testing is much less likely to be variable. There are three limitations to our study. First, we generated our findings from a relatively small number of individuals, which should be validated in larger cohorts of Chinese Han patients. Second, this study is limited by its retrospective nature and its confinement to a single center. In the future, the sample size will be expanded, or multicenter studies will be performed for further validation. Third, in this study, only associations between ART pregnancy outcomes and basal FSH/LH and OSI were investigated without addressing other confounders’ impacts.

## Conclusions

Firstly, in individuals with normal AMH levels, we observed that an increase in basal FSH/LH leads to a reduced ovarian response to exogenous Gn. Secondly, the OSI exhibited a strong correlation with female parameters associated with ovarian reserve. Thirdly, we identified threshold effects for basal FSH/LH and OSI in both normal and low anti-Müllerian hormone populations, with turning points at 3.5 and 2.3, respectively. Additionally, the ovarian sensitivity index (OSI) independently impacted the ovarian response. These findings could assist clinicians in evaluating ovarian response in patients with normal AMH levels undergoing assisted reproductive technology (ART) treatments for infertility. By employing factor analysis, we may be able to better understand the underlying relationships among variables like AMH, AFC, FSH/LH, and others, and potentially reveal novel patterns or factors that contribute to ovarian reserve and response. Consequently, further research is required to elucidate the relevant underlying mechanisms.

## Data availability statement

The original contributions presented in the study are included in the article/supplementary material. Further inquiries can be directed to the corresponding authors.

## Ethics statement

The study was approved by the Ethics Committee of the Affiliated Hospital of Southwest Medical University (ethics code number: KY2022300). Informed consent from study participants was not required.

## Author contributions

XS and CY: Conceptualization. XS, LL, FY, and CS: Data curation, Writing-Original draft preparation. YH, MM, and YL: Revising the manuscript critically for important intellectual content. All authors agree with the contents of the manuscript.
